# The Skin and Inflamm-Aging

**DOI:** 10.3390/biology12111396

**Published:** 2023-11-02

**Authors:** Rashi Agrawal, Anne Hu, Wendy B. Bollag

**Affiliations:** 1 Department of Medicine, Medical College of Georgia at Augusta University, Augusta, GA 30912, USA; raagrawal@augusta.edu (R.A.); ahu@augusta.edu (A.H.); 2 Charlie Norwood VA Medical Center, Augusta, GA 30904, USA; 3 Department of Physiology, Medical College of Georgia at Augusta University, Augusta, GA 30912, USA

**Keywords:** aging, atopic dermatitis, epidermis, epidermal barrier, inflammation, keratinocytes, psoriasis, skin, xerosis

## Abstract

**Simple Summary:**

Aging affects all cells of the body, leading to impaired system function. Although scientists are beginning to identify some mechanisms underlying the aging process, a complete understanding remains elusive. One characteristic of aged individuals is chronic low-level inflammation throughout the body. Since inflammation induces oxidative stress and other effects that can impact proper system functioning over time, this chronic inflammation is thought to promote aging, called inflamm-aging. The cause of inflamm-aging is unclear but likely involves immune system malfunction and various other age-related alterations. Excessive inflammation of the skin, the largest organ of the body, can result in widespread effects on other systems, as is seen with inflammatory skin diseases such as psoriasis and atopic dermatitis. An important function of skin is to serve as a barrier to prevent the entry of environmental insults, such as microorganisms, and to retain water and other important substances inside the body. Disruption of this barrier results in skin inflammation that can impact the whole individual. It is known that, with age, our skin becomes less able to maintain the barrier. Therefore, we propose and discuss evidence for the idea that a poorly functioning skin barrier contributes to inflamm-aging.

**Abstract:**

With its unique anatomical location facing both the external and internal environment, the skin has crucial functions, including shielding the body from damage caused by ultraviolet radiation and chemicals, preventing water loss, acting as a primary barrier against pathogens, participating in metabolic processes like vitamin D production and temperature control and relaying information to the body through sensory and proprioceptor nerves. Like all organ systems, skin is known to undergo multiple changes with aging. A better understanding of the mechanisms that mediate aging-related skin dysfunction may allow the creation of targeted therapeutics that have beneficial effects not only on aged skin but also on other organs and tissues that experience a loss of or decline in function with aging. The skin is the largest organ of the body and can contribute to serum inflammatory mediator levels. One alteration known to occur with age is an impairment of skin barrier function; since disruption of the barrier is known to induce inflammation, skin may be a major contributor to the sustained, sub-clinical systemic inflammation associated with aging. Such “inflamm-aging” may underlie many of the deleterious changes observed in aged individuals. This review explores the role of age-related skin changes, skin inflammation and inflamm-aging.

## 1. Introduction

Aging affects all cells, tissues and organs in the body and leads to altered, deficient and/or a loss of function in these systems through multiple mechanisms. One hallmark of aging is persistent, low-level systemic inflammation; this inflammation, termed inflamm-aging, is thought to contribute to many detrimental effects of aging. The skin interacts with both the external environment and the internal milieu and functions as a barrier to block the entry of harmful stimuli while retaining important substances inside the organism. Since the skin is the largest organ of the body, disruption of this barrier leads not only to skin inflammation but also systemic inflammation. Such systemic inflammation also characterizes many inflammatory skin diseases. This review discusses accumulating evidence to indicate that deficiencies in the skin barrier during aging also lead to widespread inflammation and may be an important contributor to inflamm-aging. The intention is not to provide an exhaustive report of all that is known about aging, inflamm-aging and the skin; the goal is instead to present information supporting the involvement of skin and its permeability barrier in preventing systemic inflammation and to describe how changes in this barrier with age can impact the entire organism. 

Skin, along with the epithelia of the oral cavity, gastrointestinal tract and kidney, serves as a vital interface between the internal milieu of the human body and the external environment. Beyond its fundamental role as a physical barrier, the skin performs a multitude of complex functions that contribute to maintaining homeostasis through the relay of important sensory inputs, the regulation of temperature control and the production of important biomolecules [1]. Comprising three layers that traverse the entire body (the epidermis, dermis and hypodermis), the skin is a dynamic and complex organ with an intricate structure and cell types that work in harmony. 

**Figure 1:**
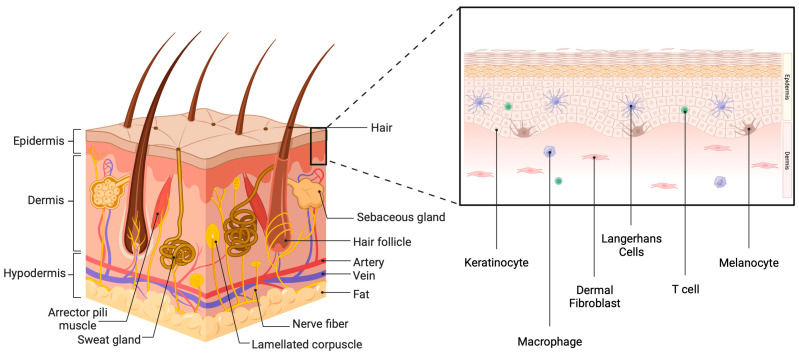
The skin. The skin comprises three layers: the epidermis, the dermis and the hypodermis. In addition to vasculature and nerve fibers, the skin also contains skin appendages, such as sweat glands, sebaceous glands and hair follicles. There are also corpuscles (Merkel cells) that allow sensation. The epidermis is also composed of multiple layers and contains several cell types. Approximately 90% of epidermal cells are keratinocytes, with interspersed melanocytes, Langerhans cells and T cells. The dermis comprises connective tissue containing fibroblasts. Created with Biorender.com.

The most superficial layer, the epidermis, is composed of five (thick skin, such as of the palms and soles) or four (thin skin) subdivisions: the strata corneum, lucidum (in thick skin only), granulosum, spinosum and basale. The stratum corneum is composed of lamellar lipids surrounding terminally differentiated, dead keratinocytes that are “armored” with cornified envelope proteins cross-linked by transglutaminases to produce mechanically strong squames. Together, the squames and lipids generate a mechanical and water permeability barrier that both excludes potentially damaging environmental insults and prevents the loss of water [2]. In thick skin, beneath the stratum corneum lies the translucent stratum lucidum. Under the stratum lucidum, or stratum corneum in thin skin, is the stratum granulosum, which provides structural reinforcement and acts as an additional water barrier. The lipids that form the water permeability barrier are produced in the stratum granulosum, packaged into lamellar bodies together with lipid-metabolizing enzymes and released into the stratum corneum as cells undergo terminal differentiation through programmed cell death. The next layer, the stratum spinosum, comprises layered spinous keratinocytes that have initiated (closest to the stratum basale) or are progressing in (as they move up through the layers) their differentiation process, undergoing cell arrest and expressing differentiation markers, such as the mature keratins, keratin 1 and 10. The deepest layer of the epidermis is the stratum basale, a single layer of cuboidal or columnar cells resting on the basement membrane and expressing the immature keratins, keratin 5 and 14, that serve to provide mechanical strength to the basal keratinocytes. Additionally, the stratum basale contains epidermal-resident dendritic cells, Langerhans cells (LCs) and T cells, as well as melanocytes that produce melanin, responsible for skin pigmentation [3]. The dermis comprises collagen fibers and dermal fibroblasts and includes papillae, projections of the dermis that house blood vessels and extend into the epidermis to provide nutrients and tactile sensation. The third and final layer of the skin is the hypodermis, also known as subcutaneous fat tissue, which is rich in adipocytes, proteoglycans and glycosaminoglycans [4] (Figure 1).

As previously mentioned, the skin plays a pivotal role in mediating immune responses to bolster the body’s defense mechanisms. The immune system’s primary role is to protect the body against any external aggressors. The inflammatory cascade serves the purpose of eliminating harmful stimuli and repairing damage. This cascade can be initiated through two pathways: the innate or the adaptive immune system. Innate immunity mediates the inflammatory process through cellular players such as neutrophils, macrophages and natural killer cells (NK cells) to recognize pathogen-associated molecular patterns (PAMPs), microbial components that interact with pattern recognition receptors to alert innate immune cells to danger. The adaptive immune response relies on B cells, T cells and circulating antibodies to establish pathogen memory over time. Activation of the inflammatory response involves the expression of pro-inflammatory markers and suppression of anti-inflammatory genes, leading to the initiation of intracellular pro-inflammatory cascades. This ultimately leads to a system-wide “danger” response known as systemic inflammation [5]. 

Although the skin is traditionally considered part of the innate immune system through its service as a physical barrier, it also exhibits adaptive immune characteristics. As noted above, the stratum basale of the epidermis contains both T cells and LCs, which play a role in the inflammatory cascade [6]. T cells have the dual ability to assist B cells in creating antibodies and recruiting neutrophils (CD4+ T cells), as well as directly killing pathogen-infected cells (CD8+ T cells). Under inflammatory conditions, LCs respond by producing pro-inflammatory mediators, serving as a liaison between the innate and adaptive immune system [4]. In addition, keratinocytes have been reported to express major histocompatibility complex II and present antigens to T cells under certain conditions [7,8]. Together, various cells residing in the skin act to protect the organism from environmental insults and external invaders. However, this protective function can be compromised by injury and disease processes, as well as by age.

## 2. Mechanisms Underlying the Aging Process

With the passage of time, all organs and tissues show signs of age-dependent impaired function. This can manifest as an increased risk for age-related diseases, such as cancer and diabetes, or reduced function, for instance, with muscle frailty or impaired skin wound healing (reviewed in [9,10]). Although all tissues experience aging, certain insults can accelerate the process. For example, exposure to solar ultraviolet radiation can induce sun-exposed skin to demonstrate a more pronounced aged appearance, although even non-exposed skin is subject to chronological aging [10]. Exposure to carcinogens in tobacco smoke can also promote an earlier predisposition to age-related skin disorders [11]. Certain clinical conditions may also hasten skin tissue dysfunction. 

**Figure 2:**
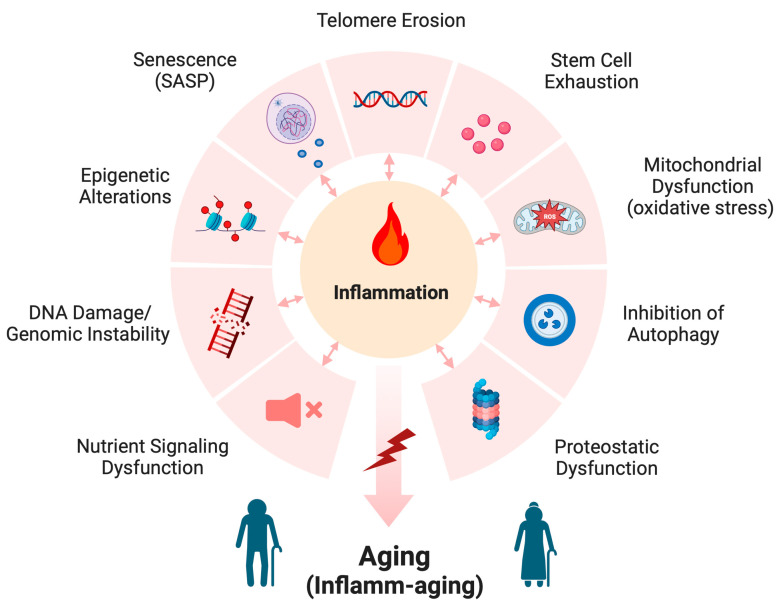
Mechanisms of aging. The mechanisms thought to underlie the aging process include proteostatic dysfunction, inhibition of autophagy, mitochondrial dysfunction (which results in increased oxidative stress), stem cell exhaustion, telomere erosion, senescence (and the associated senescence-associated secretory program, or SASP), epigenetic alterations, DNA damage and genomic instability, and nutrient signaling dysfunction. These various mechanisms often increase inflammation, which leads to inflamm-aging. Modified from [15,21] and created with Biorender.com.

Although aging is inevitable, the underlying processes are unclear. Numerous scientists have proposed a variety of mechanisms thought to mediate the aging process, the so-called pillars of aging. Many of these pillars also contribute to the pathogenesis of disease. As illustrated in Figure 2, these include (1) proteostatic dysfunction that can lead to protein aggregation and accumulation, disrupting normal cell processes, and (2) the possibly related inhibition of autophagy. Reduced autophagy not only can inhibit the clearance of these abnormal proteins but also can render mitochondria inefficient (if mitophagy is also affected and defective mitochondria are not destroyed), resulting in (3) mitochondrial dysfunction. Mitochondrial dysfunction, in turn, results in the enhanced generation of reactive oxygen species (ROS) and increased oxidative stress. ROS molecules contribute to cellular toxicity and inflammation via reactivity with proteins (often through cysteine residues), lipids (i.e., lipid peroxidation) and nucleic acids (described later). In turn, these modified macromolecules can further enhance oxidative stress, e.g., by activating a DNA damage response or, in the case of lipid peroxidation and the formation of 4-hydroxynonenal (4-HNE), through activation of the pattern recognition receptor, toll-like receptor 4 (TLR4), and the transcription factor, nuclear factor kappa-light-chain-enhancer of activated B cells (NF-κB) [12]. NF-κB can also be both activated and suppressed directly by ROS and oxidative stress, depending on the cell type and experimental conditions [13]. NF-κB is known to induce the expression of pro-inflammatory cytokines, which can promote increased oxidative stress through the recruitment and activation of immune cells, as well as enzymes that produce pro-inflammatory endogenous lipid mediators, such as various prostaglandins and leukotrienes [12].

In addition, there is evidence that (4) stem cells, multipotent cells able to regenerate the various cell types comprising the body, become exhausted and incapable of continued tissue maintenance. Although the etiology of this exhaustion is unknown, it may possibly relate, at least in part, to the (5) erosion of telomeres, which are DNA sequences on the ends of chromosomes that cap them and prevent them from sticking to one another. With each replication of the chromosomes during cell division, these telomeres shorten and eventually no longer properly cap the chromosome ends. Once their telomeres are reduced to a certain length, cells withdraw from the cell cycle; although these cells continue to be metabolically active, they no longer divide, a state termed (6) senescence, which is also increased in aged individuals [14]. Senescent cells often express a senescence-associated secretory program (SASP) [15,16], which is characterized by the release of a variety of cytokines and other inflammatory mediators that are thought to contribute to inflamm-aging, as discussed below (and please see Figure 2). Conversely, pro-inflammatory signals have been shown to promote senescence as well (e.g., [17]). 

Aging is also associated with changes in gene expression as a result of (7) epigenetic changes such as DNA and histone methylation, histone acetylation and differences in the expression of microRNAs and long non-coding RNAs, among other epigenetic modifications [9,16]. Changes in gene expression can also be a consequence of (8) DNA damage (arising from, e.g., irradiation or oxidative stress) and/or genomic instability, particularly when important proto-oncogenes, tumor suppressors or DNA repair mechanisms are mutated; these mutations can also lead to the dysfunction of other cellular processes with aging. In addition, (9) alterations in nutrient sensing also occur with aging such that anabolic signaling induced by feeding promotes age-related changes; conversely, caloric restriction, resulting in reduced signaling, extends lifespans [15]. Many of these changes seen with age can also activate the immune system to stimulate inflammation. Indeed, many models of aging, including aged humans, exhibit elevated serum levels of various cytokines and other inflammatory mediators [18,19,20]. This chronic low-level inflammatory response observed in aged individuals has been termed inflamm-aging, which is thought to be an important contributor to the aging process.

## 3. Skin Inflammation

### 3.1. Skin Inflammation following Barrier Disruption

**Figure 3:**
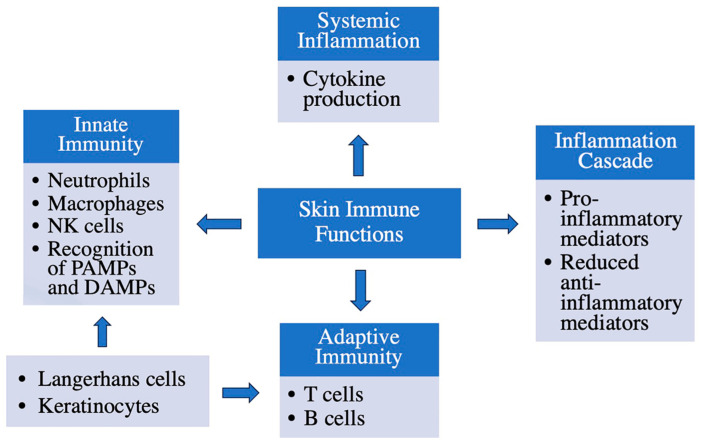
Immune functions of the skin. The skin has several important immune functions: in addition to forming a barrier to protect against microbial invasion, keratinocytes play a role in sensing and responding to environmental insults, as do Langerhans cells, resident dendritic cells. Resident T cells, macrophages and natural killer (NK) cells also monitor the tissue for potential harmful actors. Many of these cells can be activated by microbial products (pathogen-associated molecular patterns, or PAMPs) or endogenous molecules released by endangered or damaged cells (danger- or damage-associated molecular patterns, or DAMPs). In addition, these cells can recruit other immune cells into the skin to mount an immune response. Thus, infiltrating neutrophils, monocytes/macrophages and T and B cells may also participate in the response of the immune system.

The initial school of thought regarding inflammation in the skin, especially with regard to common cutaneous skin disorders such as atopic dermatitis and psoriasis, was that the epidermis and epidermal keratinocytes are the targets of inflammatory mediators circulating in the body from another source separate from the skin itself [22]; however, further investigation demonstrated that the skin can serve as the initiation site of these inflammatory responses. LCs, epidermis-resident antigen-presenting (dendritic) cells, are an important factor underlying immune system responses and inflammation of the skin, but epidermal keratinocytes are also able to express and secrete pro-inflammatory cytokines [23]. Skin immune functions and the cells involved are summarized in Figure 3. Importantly, inflammation in the skin can also lead to systemic inflammation [18,20,24,25] (see below).

One factor that can influence skin inflammation is acute disruption of the cutaneous barrier [26], which is, in fact, a common initiating cause of skin inflammation [27]. As noted above, the epidermis of the skin is composed primarily of keratinocytes, with basal keratinocytes attached to the basement membrane at the dermal–epidermal junction. These basal cells continuously proliferate to regenerate the epidermis and replace cells that are sloughed to the environment (reviewed in [10,28]). As basal keratinocytes lose their attachment to the basement membrane, they progress to the next layer, the spinous layer, and initiate a differentiation program that eventually culminates in cell death and the formation of the outermost layer of the epidermis, the stratum corneum or cornified layer. The stratum corneum consists of dead keratinocyte “bricks” and a lipid “mortar” that together create a mechanical and water permeability barrier that protects from external insults and maintains the internal milieu [10,28]. When this epidermal permeability barrier is acutely disrupted in young mice, a rapid increase in the mRNA and protein expression of various cytokines in the skin results [26], as well as epidermal hyperplasia and thickening [29]. Concomitantly, these mice exhibit elevated levels of cytokines in the bloodstream [18]. 

LCs are also affected in terms of their maturation and inflammatory mediator response upon acute permeability barrier disruption. Using a hairless mouse model, Katoh et al. [30] assessed the levels of expression of MHC Class II, B7-1, B7-2 and intercellular adhesion molecule (ICAM)-1 upon acute barrier disruption; these co-stimulatory molecules act together to trigger an immune response from T cells. Interleukin (IL)-1β levels were also examined since IL-1β is an inflammatory molecule released by LCs. These authors found that there was a significant increase in the levels of MHC Class II, B7-2 and ICAM-1 along with upregulation of the levels of IL-1β in the 12 h after epidermal barrier disruption [30]. Furthermore, another study examining the effects of occlusion on the levels of epidermal pro-inflammatory cytokines, such as IL-1α, IL-1β, tumor necrosis factor (TNF)-α and granulocyte-macrophage colony-stimulating factor, found that these inflammatory mediators are also increased by epidermal barrier disruption [31]. 

### 3.2. Inflammatory Skin Diseases

Atopic dermatitis (AD) is a common eczematous skin disorder that is characterized by skin inflammation and barrier disruption, often resulting in chronic pruritis and marked excoriations. Due to its root cause of inflammation and its widespread prevalence, AD provides researchers more insight into how various pro-inflammatory cytokines and other mediators interact and behave in the human epidermis. Recent evidence suggests that IL-4 and IL-13 play a significant role in the development of AD. IL-4 and IL-13 share a common receptor and signaling pathway but are expressed differently by various cells involved in innate and adaptive immunity. For instance, IL-4 is expressed by lymph node T follicular helper cells (Th2), basophils and invariant natural killer T2 cells. On the other hand, IL-13 is predominantly expressed by mucosal group 2 innate lymphoid cells. Transcriptomic analysis has revealed that IL-13 has a specific and dominant role in skin lesion development associated with AD [32]. IL-13 signaling is also associated with an enhanced itch sensation and impaired skin barrier function due to the inhibition of molecules involved in skin differentiation. It also stimulates the production of chemokines [chemokine (CC motif) ligand (CCL)17 and CCL22] that recruit chemokine receptor-4+ (CCR4+) Th2 cells to the affected tissue. Additionally, IL-13 is implicated in AD-related fibrosis, as it is a profibrotic cytokine [33]. 

**Figure 4:**
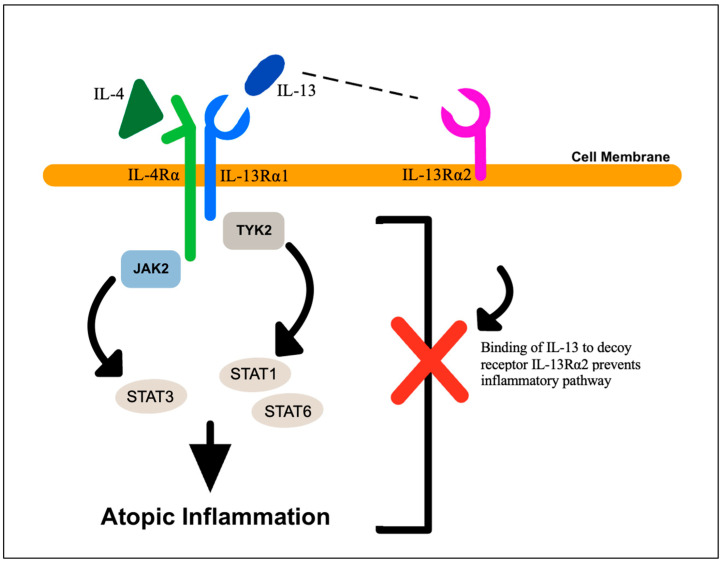
Cytokine signaling in atopic dermatitis. Atopic dermatitis lesions are characterized by elevated levels of IL-4 and IL-13, which can signal through their receptors, IL-4Rα and IL-13Rα1, respectively, to activate Janus kinase-2 (JAK2) and tyrosine kinase-2 (TYK2). These kinases phosphorylate and activate members of the signal transducer and activator of transcription (STAT) family of transcription factors, STAT1, STAT3 and STAT6. The decoy receptor, IL-13Rα2, can bind IL-13 and prevent it from binding to IL-13Rα1, thus inhibiting signaling through the JAK–STAT pathway.

In addition, the binding of IL-4 and IL-13 to the functional heterodimeric receptors IL-4Rα and IL-13Rα1 induces the activation of downstream Janus kinase 2 (JAK2) and tyrosine kinase 2 (TYK2), which further activate members of the transcription factor family, signal transducer and activator of transcription (STAT): STAT3 by JAK2 and STAT6 and STAT1 by TYK2. This signaling cascade in the epidermis is ultimately responsible for the atopic inflammation seen in AD. In addition, this signaling can be inhibited by a decoy receptor called IL-13Rα2, which binds to IL-13 and prevents activation of the JAK–STAT inflammatory signaling cascade (Figure 4). In some cases, important pro-inflammatory mediators in human keratinocytes, such as IL-13, IL-4 and TNF-α, can paradoxically decrease skin inflammation since their presence can augment and upregulate the expression of IL-13Rα2 [34]. AD provides a clinical model in which to study key factors in inflammation and to generate insight into the interaction of different inflammatory mediators in the epidermis. 

Psoriasis is another common inflammatory skin disease, mediated predominantly by the T-helper cell-17 (Th17) immune pathway [35,36,37]. IL-17 was initially known as a key cytokine in the regulation of immune responses through its effects on chemokine expression and tissue inflammation. It was later discovered that its production is mediated by a previously unknown subgroup of antigen-specific effector CD4+ T cells named Th17 cells [38]. Differentiation of these specific helper T cells is distinct from the development of Th1 or Th2 cells [35]. Using T cell clones from lesional psoriatic skin, IL-17 has been shown to stimulate human keratinocytes and preferentially increase the production of IL-6 and IL-8, which are potent pro-inflammatory cytokines; this effect is augmented by interferon-γ, which is also present in infiltrating T cells of inflamed psoriatic skin [36]. The correlation between IL-17 (derived from Th17 cells) and the inflammation present in psoriasis is also seen with another inflammatory mediator, IL-25, a member of the IL-17 cytokine family that is also significantly expressed in psoriasis lesions [39]. Xu et al. [37] were able to demonstrate the involvement of this cytokine in the pathogenesis of psoriasis by inducing psoriasis-like skin lesions with an injection of IL-25. This result, coupled with the finding that the ablation of the IL-25 gene in a transgenic mouse model significantly reduces epidermal hyperplasia in and dermal immune cell infiltration into psoriasis-like skin lesions [37], shows the importance of this cytokine in psoriasis [39]. Although AD and psoriasis show some differences in the relationship between inflammation and the pathogenesis of disease, they provide a background for which to study key factors in inflammation and allow more insight into the interaction of different inflammatory mediators with and within the epidermis.

### 3.3. Inflammatory Skin Diseases and Systemic Inflammation

Although the previous section mainly focuses on the mechanism of inflammation in the skin, it should be noted that, in diseases like psoriasis and AD, skin inflammation can contribute to systemic increases in inflammatory mediator levels [18,20]. For example, a meta-analysis found that IL-6 and TNFα, as well as C-reactive protein and ICAM-1, were higher in patients with psoriasis compared to healthy controls [24]. Another study demonstrated a correlation between AD severity and the blood levels of several pro-inflammatory markers, as well as the lesional mRNA expression of some of these same markers [25]. In turn, these increases in systemic inflammatory mediators are thought to provide the basis for findings in various studies [40,41,42], according to which psoriasis and AD are associated with an increased risk for certain cardiovascular disorders. It should also be noted that both AD and psoriasis are characterized by disruption of the epidermal permeability barrier, which, as mentioned previously, leads to skin inflammation [18,20]. In fact, the degree of barrier disruption for both AD and psoriasis correlates with the severity of the disease [27], and in some cases, particularly for AD, this disruption is thought to underlie disease development [27]. For instance, genome-wide association studies have found a link between the important barrier protein filaggrin and AD [43], and it has been suggested that an impaired barrier contributes to the development of the disease (reviewed in [44]). 

Pondeljak and Lugović-Mihić [45] reviewed the skin environment in reference to stress-induced interactions between immune cells, neurotransmitters and hormones. Specifically, these authors focused on keratinocyte-derived cytokines and chemokines—most notably IL-1 and IL-6—which are able to function in an autocrine or paracrine manner, with effects on inflammatory cell migration, keratinocyte proliferation/differentiation and the production of other cytokines. As a result, they can have systemic impacts on the immune system. During acute stress, the central nervous system (CNS) experiences pro-inflammatory effects, as stress increases the permeability of the blood–brain barrier to mast cells. Consequently, IL-6 can potentially cross the compromised blood–brain barrier and stimulate the hypothalamic–pituitary–adrenal (HPA) axis [46]. Furthermore, IL-6 can promote lymphocyte activation, boost antibody production through CD4+ T helper cells and trigger fever and acute-phase reactant synthesis [47]. 

There are other keratinocyte-derived cytokines, particularly IL-10 and IL-12, which also have widespread effects. For instance, IL-10 is responsible for immunosuppression by shifting the cytokine profile away from T helper cells, specifically Th1 and Th2, thus suppressing immune reactions. IL-12, secreted by both normal and transformed human keratinocytes, induces a systemic response mediated by Th1 immune effectors [48]. The effects of IL-10 and IL-12 are inverse, and their production seems to be influenced by the duration of psychological stress. During short-term stress exposure, the body experiences increased IL-12 secretion, which promotes inflammation. On the other hand, the secretion of anti-inflammatory IL-10 secretion is enhanced during chronic stress [49], perhaps in an attempt to mitigate the effects of chronic inflammation. In summary, inflammation is both a local and systemic response of the body to a wide variety of stressors, and skin seems to both respond to and contribute to stressful stimuli.

## 4. Skin Aging

### 4.1. Factors Involved in Skin Aging

**Figure 5:**
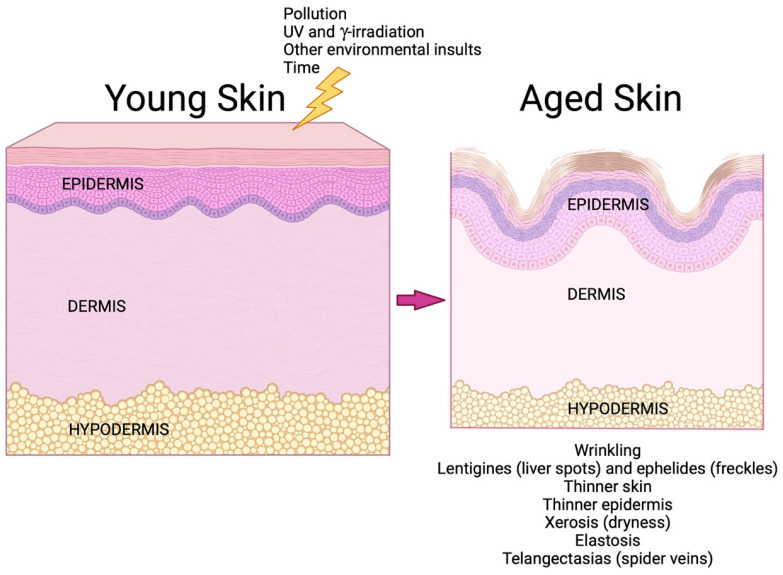
Skin aging. Over time, aging skin undergoes a number of alterations that contribute to dysfunction. In addition to chronological aging, certain external stimuli can accelerate aging; these include ultraviolet (UV) and γ-irradiation and exposure to pollution and other environmental insults, such as cigarette smoke [11,50]. Skin function declines with age. Reduced extracellular matrix proteins in the dermis result in wrinkling, elastosis and thinner skin (since the dermis represents 90% of the skin thickness [52]), and abnormalities in pigmentation lead to lentigines (liver spots) and ephelides (freckles) [10]. Epidermal dysfunction results in a thinner epidermis (due to decreased proliferation of aged epidermal keratinocytes) and xerosis (from reduced production of proteins involved in skin hydration and deficiencies in the epidermal permeability barrier) [53]. Telangectasias and thinning of the subcutaneous fat (in some but not all regions of the body) [11,54] also characterize aged skin. Created with Biorender.com.

Despite its multifunctionality and integral role in immune responses, the skin is not impervious to various external hazards nor to the effects of time. Skin aging can occur through extrinsic (external insults) and intrinsic (chronological) processes, with inflammation often being implicated. Extrinsic aging can result from various damaging factors, including ionizing radiation, alcohol intake, poor nutrition, pollution and ultraviolet (UV) exposure [50,51] (Figure 5). UV damage can lead to damage to the dermal extracellular matrix, as well as to keratinocyte DNA; such damage is responsible for the formation of skin defects such as wrinkles, pigmented lesions, patchy hypopigmentation and actinic keratoses [10]. 

UV radiation also triggers skin inflammation, which can often be visibly seen in terms of acute erythema and edema—commonly known as sunburn. UV irradiation of the epidermis results in the production of ROS in keratinocytes, leading to oxidative stress, which can cause mitochondrial and cell damage [55]. In an unfortunate cycle, this oxidative stress induced by UV radiation leads to heightened production of ROS, which contributes to the initiation of inflammation and the activation of pro-inflammatory cytokines such as IL-2, IL-6 and TNF-α [56]. This process involves multiple pathways, including the induction of gene expression through transcription factors such as NF-κB, hypoxia-inducible factor 1-alpha (HIF-1α), nuclear factor erythroid 2-related factor 2 (Nrf-2) and activator protein 1 (AP-1). UV radiation also activates pathways involving lipoxygenase and cyclooxygenase [57], the activity of which produces both ROS [58] and lipid mediators that induce inflammation and further enhance oxidative stress [12]. Inflammation induced by UV radiation is also associated with a deficiency of klotho, a transmembrane protein and anti-aging hormone that plays a protective role against various stressors [1].

On the other hand, intrinsic aging primarily results from the passage of time and the accumulation of ROS over time, potentially influenced by genetic factors. The mechanism of the increased ROS is not completely understood but is thought to involve cumulative mitochondrial DNA damage, leading to mitochondrial inefficiency and electron leak [59]. ROS, along with decreased protective anti-oxidant, growth factor and hormonal activity, contribute to skin deterioration. In addition to the damaging effects of ROS, decreased activity of these factors due to aging may lead to skin damage through a subsequent increase in cytokines. Intrinsic damage of the skin may manifest as thinning of the skin, changes at the epidermal–dermal junction and a reduction in melanocytes and LCs [60]. 

### 4.2. Changes in Skin Function with Age

Skin aging, both intrinsic and extrinsic, is associated with impairments in skin function, encompassing a wide array of detrimental changes. In essence, all functions of the skin are diminished with age. Accordingly, thermoregulation is compromised, as there is dysfunction of both the cutaneous vasculature, which helps to dissipate heat to the environment, and the sweat glands, which remove heat by producing sweat that allows evaporative heat loss [10]; in addition, aged skin shows a reduced number of blood vessels. Together, these effects lead to heat intolerance in older individuals. Furthermore, the dermis thins with age and shows reduced extracellular matrix production, as well as increased fragmentation and disorganization of matrix components, particularly with photoaging [10]. There also seem to be decreases in skin innervation, with reduced nerve fiber endings in the dermis and epidermis leading to diminished sensory perception [10]. The hypodermis is affected as well, with age resulting in the redistribution of adipose tissue to alter the subcutaneous-to-visceral-fat ratio and the reallocation of fat among different subcutaneous facial compartments [10]. Finally, both the epidermis and cutaneous immunity are affected by aging. An aged epidermis exhibits decreased regenerative capacity and hydration, impaired barrier homeostasis, reduced sebum and glycerol levels, an elevated surface pH [61], impaired tanning and dyspigmentation [10]. Skin immunity is depressed with aging, with age-related depletion of LCs, as noted above, and aberrant immune cell trafficking [62]. Thus, no skin cell type appears to escape the ravages of aging.

Because the skin atrophies with age, as noted above, fragility, poor wound healing and an increased risk of skin tears accompany aging of the dermis and epidermis [63]. This disorder was termed dermatoporosis by Kaya and Saurat [63] to emphasize the vulnerability of aged skin to chronic fragility. Although the incidence of dermatoporosis is unclear, a few studies have suggested that more than 30% of individuals aged 60 or older may have the condition [64,65,66]. The primary risk factor for the disorder is age, and aged skin exhibits complications related to thinning and fragility, as well as an enhanced risk for impaired wound healing and frank non-healing wounds [65]. Non-healing wounds are a major medical and financial burden on the healthcare system of the United States, affecting approximately 6.5 million patients and costing a reported 25 billion dollars or more annually in treatment expenses [67], and therapies to improve the function of aged skin are critically needed. Epidermal thinning, fragility and poor wound healing can result from impaired keratinocyte proliferation [65]. Indeed, aged skin in mice and humans shows a diminished proliferative capacity that is concomitant with reduced epidermal thickness [68,69,70,71,72,73]. Thus, despite its adaptability, continuous exposure to toxins and other environmental insults, in addition to the passage of time, compromise the skin’s homeostasis, accelerate its aging and impair its integrity and function.

Another sign of skin dysfunction in aging is the prevalence of skin disorders, which are very common in older populations [11,53]. In fact, a recent study from Finland determined that more than 75% of subjects older than 70 years of age exhibited some type of skin disease, requiring follow-up [74]. The most common skin finding was benign skin tumors, but more than 20% exhibited asteatotic eczema, also called xerosis, which is characterized by pruritic (itchy), dry and cracked skin [74]. Xerosis is associated with aging, with an estimated prevalence between 30 and 75% in nursing homes and long-term care residences [75]. Dry skin is thought to result from a reduction in the hygroscopic molecules produced by aged skin as well as a loss of moisture from the skin as a result of a dysfunctional barrier [50,61]. In turn, xerosis is the single most common cause of pruritus in older individuals [75], most likely because the reduced functionality of the epidermal barrier leads to skin inflammation, as previously discussed. In turn, scratching the itch can lead to further barrier disruption/damage to the skin and potentially to the introduction of microorganisms, which, in a vicious cycle, leads to greater inflammation and more pruritus. Indeed, pruritus is a prevalent skin condition in older individuals. Thus, in a recent study of randomly sampled United States veterans receiving Veterans-Administration-based healthcare, with a mean age of 61 years, almost 38% reported chronic pruritus [76]. Chronic pruritus can result in poor sleep quality and is associated with depression and anxiety [76]. Therefore, research to understand the factors that contribute to skin impairments with aging, as well as investigations to identify a treatment to improve aged skin function, would be of great benefit to older individuals for a multitude of reasons.

### 4.3. Possible Mechanisms of Skin Dysfunction with Age

**Figure 6:**
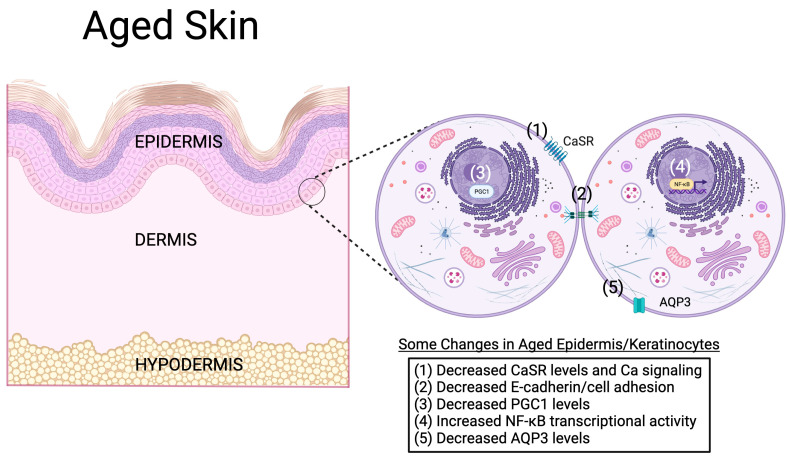
Possible molecular mechanisms of keratinocyte and epidermal aging. In the aged epidermis, a number of molecular changes have been observed. For example, aging keratinocytes in the epidermis have been found to express lower levels of the calcium-sensing receptor (CaSR), leading to impaired calcium signaling and reduced E-cadherin levels and cell adhesion [95]. The levels of the transcriptional co-activator peroxisome proliferator activator-γ co-activator-1 (PGC1) are also altered with age, and the genetic knockdown of PGC1 expression reproduces characteristics of aged keratinocytes/epidermis [77,81]. On the other hand, the transcriptional activity of the transcription factor, nuclear factor kappa-light-chain-enhancer of activated B cells (NF-κB), is increased with age [13]. The ability of NF-κB to induce the expression of inflammatory mediators might underlie chronic low-level inflammation associated with aging. Finally, the mRNA and protein expression of the aquaglyceroporin, aquaporin-3 (AQP3), is reduced in aged skin [88,89,90]. Because evidence supports an important role for AQP3 in keratinocyte proliferation, differentiation and migration, as well as skin hydration, wound healing and permeability barrier function [28], decreased levels of this protein might mediate, at least in part, age-related skin changes. Created with Biorender.com.

Although the mechanism(s) underlying the reduced proliferative capacity of the epidermis with aging is unclear, research is beginning to uncover some potential etiologies, as illustrated in Figure 6. For example, a recent study investigated the effect of aging on the expression of peroxisome proliferator activator-γ coactivator-1 (PGC1α) in mouse skin. This study showed reduced epidermal PGC1α levels with aging; in addition, this decrease was associated with delayed wound healing in aged mice [77], suggesting a possible role for this co-activator in aging-related skin changes. Indeed, a conditional knockout mouse model with an epidermal-specific ablation of the gene encoding PGC1α exhibited decreased keratinocyte proliferation and delayed wound healing, demonstrating the importance of this protein to skin function [77]. These results support the potential involvement of PGC1α in some (or perhaps all) of the deficiencies associated with age. This idea is bolstered as well by the fact that exercise is known to have positive effects on both skin structure and function; exercise increases skin hydration [78], reduces the thinning of the stratum corneum observed with aging [79] and accelerates skin wound healing [80]. Since exercise also increases PGC1α levels in the skin [79], it is possible that the positive effects of exercise on aged skin may also be mediated by this co-activator. On the other hand, using immunohistochemistry, Gravel et al. [81] reported that, in human skin, PGC1α protein levels increase with aging. Nevertheless, these authors also showed that the knockdown of PGC1α and PGC1β reduces the proliferation of human keratinocytes and results in the generation of a thinner reconstructed living human epidermal equivalent in a three-dimensional culture [81].

Another potential player in the changes in proliferative capacity observed in aged skin is the aquaglyceroporin, aquaporin-3 (AQP3). AQP3, the most abundant aquaporin (water pore) in the epidermis, is a channel that transports water, glycerol and other small molecules [28]. Verkman and colleagues have demonstrated a pro-proliferative role for AQP3 in both mouse and human keratinocytes [82,83,84,85]. Thus, the knockdown of AQP3 in human keratinocytes decreases proliferation, and in AQP3 knockout mouse keratinocytes, AQP3 deficiency results in a decreased cellular ATP content and impaired proliferation [83]. Data from AQP3 knockout mice also provide evidence for an important role of AQP3 in vivo, as these mice exhibit a reduced water holding capacity, decreased elasticity and delayed water permeability barrier repair after disruption [86,87]. Consistent with a possible role for AQP3 in skin aging, AQP3 is decreased in both extrinsically (sun-exposed) and intrinsically (chronologically) aged human skin [88,89]. Since AQP3 has been found to be an important contributor to skin hydration [86,87,90], these data suggest that reductions in AQP3 levels might underlie xerosis observed in aged human skin (see below). Finally, aged mice also have reduced AQP3 levels in the epidermis in comparison with younger mice [90], and in harmony with these data, AQP3 knockout mice exhibit delayed skin wound healing [87]. Together, these results suggest the potential involvement of AQP3 in skin aging.

Decreased AQP3 levels with aging might also underlie another common issue observed in aged skin: skin dryness (xerosis) and impaired permeability barrier repair following disruption [61]. Indeed, as indicated above, AQP3 knockout mice are characterized by delayed permeability repair [87]. Conversely, the over-expression of AQP3 in the epidermis (under the control of the keratin-1 promoter) accelerates epidermal barrier repair [91]. In AQP3 knockout mice, AQP3 deficiency is associated with a reduced epidermal (stratum corneum) glycerol content [92], and in fact, restoration of epidermal glycerol levels with pharmacologic doses of glycerol improves the epidermal phenotype observed in these mice [92]. Glycerol, in turn, is known for its hygroscopic (water-attracting) properties, underscoring the likely importance of AQP3 for skin hydration [86]. Notably, skin (stratum corneum) hydration has also been reported to inversely correlate with inflammation and serum TNFα levels [93]. Therefore, the decrease in AQP3 seen with aging may impact skin function through multiple mechanisms, e.g., reduced glycerol content [61], poor skin hydration, enhanced inflammation and impaired keratinocyte proliferation and permeability barrier repair.

Another pathway that has recently been shown to be altered with skin aging is activation of the keratinocyte calcium-sensing receptor (CaSR). This G-protein-coupled receptor binds calcium ions as its ligand, stimulating phosphoinositide hydrolysis to regulate keratinocyte function [94]. The presence of CaSR likely explains the ability of extracellular calcium concentrations to regulate keratinocyte proliferation and differentiation in vitro, with low calcium levels promoting proliferation and elevated calcium concentrations inducing differentiation [94]. Calcium is thought also to modulate these processes in vivo, as a calcium gradient is observed in the epidermis in situ, correlating with the effects in vitro. Thus, the calcium gradient is lowest in the basal layer and gradually increases to maximal levels in the stratum granulosum [94]. Mauro and colleagues [95] demonstrated that the levels of CaSR are reduced in aged keratinocytes/epidermis, as are those of E-cadherin, resulting in decreased keratinocyte cell–cell adhesion. Furthermore, these decreased levels result in impaired calcium signaling and keratinocyte migration [95], suggesting their likely involvement in the delayed wound healing observed in aged individuals. Since epidermal-specific CaSR knockout mice also exhibit a disrupted epidermal calcium gradient, reduced markers of differentiation and delayed permeability barrier repair [96], the aging-related reduction in CaSR levels may also underlie the skin barrier dysfunction seen with aging.

However, another pathway that might be involved in skin aging is the transcription factor NF-κB, which shows increased DNA binding with chronological age in skin and other tissues [13]. This transcription factor is important in regulating epidermal keratinocyte and immune cell function, as well as inflammatory mediator production, through its canonical signaling pathway. NF-κB is activated in response to a diverse set of stimuli, including the activation of T cell, B cell and pattern recognition receptors, oxidative stress, physical stress, DNA damage (genotoxic stress), growth factors and cytokines [13]. NF-κB also participates in a non-canonical signaling pathway that is thought to supplement the canonical NF-κB signaling pathway in the adaptive immune system [97] and to play important roles in development [13]. NF-κB has been noted to induce the activation and transcription of various pro-inflammatory genes while also regulating inflammatory T cells in terms of their activation, differentiation and effector functions [98]. The impact of NF-κB on skin inflammation can be seen in vivo in mice lacking IκBα, an inhibitory protein that controls NF-κB transcriptional activity through sequestering it in the cytoplasm. Global IκBα knockout mice, which die 7 to 10 days after birth, exhibit increased activation of the NF-κB pathway, which results in severe widespread inflammation in the skin accompanied by increased skin TNFα levels [99]. The skin of these mice is also characterized by extensive keratinocyte proliferation, epidermal hyperplasia, an abnormal granular layer and dermal/epidermal infiltration with immune cells, including corneal microabscesses [99]. A similar initial phenotype of keratinocyte hyperproliferation and epidermal hyperplasia was observed in mice with epidermal-specific conditional deletion of IκBα, although mice with skin-limited IκBα deficiency do not die and in fact show improvements in their skin disease by 3 weeks of age [100]. These mice also demonstrate an intact granular layer with no immune cell infiltration or microabscess formation [100]. Together, these results demonstrate the clear importance of NF-κB in skin inflammation, both in epidermal keratinocytes and other cell types, and suggest that the enhanced NF-κB transcriptional activity observed with aging could contribute to chronic inflammation in aged skin. In addition, NF-κB also plays a key role in senescence and in driving SASP [13,101] and, in this manner, also helps to mediate both skin inflammation and skin aging.

Finally, NF-κB can also promote oxidative stress, in part through its effects on inflammation and SASP, but also by inhibiting Nrf2 [102]. Nrf2 is a transcription factor that induces the expression of enzymes involved in endogenous anti-oxidant systems, such as glutathione peroxidase, heme oxygenase-1 and NAD(P)H quinone oxidoreductase (NQO1). For the most well-characterized mechanism, the activation of Nrf2 can be induced by oxidative stress, which results in the oxidation and inactivation of Kelch-like ECH-associated protein 1 (Keap1), an inhibitor of Nrf2 via its ability to promote Nrf2 ubiquitination and degradation (although there is a Keap1-independent regulatory mechanism as well) [102]. Oxidized, inactivated Keap1 dissociates from Nrf2, allowing this transcription factor to enter the nucleus and stimulate the expression of endogenous anti-oxidant systems. NF-κB can inhibit Nrf2 directly at the transcriptional level, in part by competing for the transcriptional co-activator cAMP response element-binding protein (CREB)-binding protein (CBP) [102]. NF-κB also inhibits PGC1α [103]. 

## 5. Inflamm-Aging

The process of aging is accompanied by the deterioration of immune functions, known as immune aging or immune senescence. Paradoxically, throughout a person’s lifetime, continuous exposure to environmental factors and interactions with infectious agents, as well as perhaps other aging-related changes such as increased senescence, contribute to a state of chronic inflammation in older individuals, referred to as inflamm-aging. This state is characterized by an increase in pro-inflammatory mediators in the bloodstream. The aging process also brings about changes in T cell balance, which are associated with a decline in immunity and an elevation in inflammation [104]. This dyshomeostasis is hypothesized to happen in part due to the increased presence of regulatory T cells impairing the effectiveness of CD8+ and natural killer cells; additionally, there may be a decrease in naïve T cells, which can result in compromised acquired immune responses [105]. Conversely, the expansion of CD25-null T cells may lead to the increased secretion of TNF-α and IL-6, thereby intensifying the degree of inflammation [106]. 

A case-controlled study evaluating serum cytokine profiles sought to quantify the inflammatory and immune system changes that occur with age. Kim et al. [107] found that soluble CD40 ligand (sCD40L) and transforming growth factor-α (TGF-α) levels were significantly higher in elderly patients when compared to a younger cohort. The CD40/CD40L system, which is part of the tumor necrosis factor superfamily, plays a crucial role in connecting inflammation and atherothrombosis. Furthermore, interactions between sCD40L and CD40 lead to elevated levels of oxidative stress and endothelial dysfunction, which can further contribute to the escalation of the inflammatory cascade and to vascular pathologies. TGF-α, which is a member of the epidermal growth factor family, is a powerful stimulator of cell division and migration. It plays a crucial role in the normal growth and development of various tissues and organs, as well as in processes like angiogenesis, wound healing and bone remodeling. Additionally, TGF-α is involved in the pathogenesis of several diseases, including coronary artery disease, cystic fibrosis, psoriasis, oral leukoplakia and cancer [107]. However, although there is evidence of increased inflammation, including higher levels of C reactive protein and altered levels of immunomodulatory molecules in aged individuals, there is no definitive answer for the basis of these changes. With this age-associated systemic inflammation and the prior knowledge that aged skin has a compromised homeostasis, it has been proposed that skin may be a major source of circulating inflammatory mediators and therefore inflamm-aging [108]. 

In experiments with mice, several important observations have been obtained in relation to the hypothesis that the skin contributes significantly to inflamm-aging. First, as noted previously, acute barrier disruption in mice induces cytokine expression in the skin as well as increased cytokine levels in circulation [26]. Aged mice with epidermal barrier dyshomeostasis also show increases in both skin and serum cytokine levels [18]. Second, the expression of TNF-α and amyloid A mRNA in the epidermis—but not in the liver—are paralleled by significant increases in serum cytokine levels [18]. Third, experimental barrier disruption induces similar elevations in epidermal and serum cytokine levels in both normal and athymic mice, indicating that T cells have a limited role in the increase in cutaneous and serum inflammatory cytokines caused by epidermal dysfunction [18]. Finally, correcting the epidermal barrier results in a significant reduction in cytokine levels in both the skin and serum of aged mice [18] and aged humans [20] (see below). 

Other researchers have also looked to the skin as the possible source of increased systemic inflammation with aging; specifically, Wolf et al. [109] analyzed the secretion of pro-inflammatory cytokines from human dermal fibroblasts of both the young and the elderly. In their study, inflammation in human skin fibroblasts was induced through either in vitro infection by cytomegalovirus (CMV) and/or stimulation by lipopolysaccharide (LPS). They found that human skin fibroblasts obtained from older individuals generated higher levels of IL-6 and IL-8 in response to CMV infection or LPS exposure when compared to cells obtained from younger individuals. The disparity was especially notable for IL-6, although there was also a significant difference observed for IL-8 after LPS stimulation [109]. These results are in accordance with previous observations in which fibroblasts undergoing senescence as a consequence of aging have heightened production of cytokines such as IL-6 and IL-8 [110].

In light of the fact that skin is the largest organ of the body, these findings collectively suggest that persistent abnormalities in epidermal function in chronologically aged skin may contribute to the phenomenon of inflamm-aging, potentially making the elderly more susceptible to the development or worsening of chronic inflammatory conditions. As individuals grow older, it is nearly unavoidable for them to experience one or more conditions that are commonly associated with the natural process of aging. These conditions include type 2 diabetes, atherosclerotic cardiovascular disease, Alzheimer’s disease, obesity, osteoporosis and sarcopenia [108]. Although the reason for this age-related susceptibility is not fully understood, inflamm-aging is becoming an increasingly popular theory. Other studies have also demonstrated that individuals diagnosed with chronic inflammatory skin conditions like psoriasis and AD also exhibit a higher prevalence of obesity, hypertension, prediabetes, type 2 diabetes and hypercholesterolemia, all of which predispose individuals to age-related chronic diseases [111].

Ye et al. [20], influenced by the convergence of the barrier changes observed in aged skin, inflamm-aging and age-related chronic diseases, conducted a pilot study investigating whether enhancing the barrier function of the epidermis in older individuals can lead to a decrease in the levels of pro-inflammatory cytokines circulating in their bloodstream. These authors found that the topical application of a barrier-repair emollient significantly improved the function of the epidermal permeability barrier and increased hydration in the stratum corneum [20]. Simultaneously, circulating levels of IL-1β and IL-6 in the treated elderly individuals returned to levels comparable to those of the young control individuals, whereas TNFα levels decreased in the treated elderly individuals to values that were not significantly different from those of the young controls [20]. Since (1) epidermal barrier homeostasis is impaired with age [20], (2) barrier dysfunction can lead to elevated serum pro-inflammatory cytokine levels [18,27], and (3) improvement or restoration of the barrier can reduce these levels in both mice and humans [18,20,112], these results suggest that treatments to improve the permeability barrier in aged individuals might be beneficial in combating inflamm-aging. 

## 6. Conclusions

Ultimately, many unanswered questions remain concerning aging, inflamm-aging and the role of the skin in this process. Skin aging, and consequently chronic inflamm-aging, is an intricate, multi-faceted process that is driven by both internal and external factors with a wide array of effectors, such as genetics, environmental mediators and immunological players. Hopefully, the information provided here can guide future research in understanding aged skin as a possible major cause of inflamm-aging and in pursuing therapeutics to prevent its damaging sequelae. 

## Data Availability

Not applicable.
